# Fossilized skin reveals coevolution with feathers and metabolism in feathered dinosaurs and early birds

**DOI:** 10.1038/s41467-018-04443-x

**Published:** 2018-05-25

**Authors:** Maria E. McNamara, Fucheng Zhang, Stuart L. Kearns, Patrick J. Orr, André Toulouse, Tara Foley, David W. E. Hone, Chris S. Rogers, Michael J. Benton, Diane Johnson, Xing Xu, Zhonghe Zhou

**Affiliations:** 1 School of Biological, Earth and Environmental Sciences, University College Cork, North Mall, Cork, T23 TK30 Ireland; 2 Institute of Geology and Paleontology, Linyi University, Shuangling Road, Linyi City, Shandong 276005 China; 3 School of Earth Sciences, University of Bristol, Queen’s Road, Bristol, BS8 1RJ UK; 4 UCD School of Earth Sciences, University College Dublin, Dublin, D04 N2E5 Ireland; 5 Department of Anatomy and Neuroscience, University College Cork, Western Road, Cork, T12 XF62 Ireland; 6 School of Biological and Chemical Sciences, Queen Mary University of London, Mile End Rd., London, E1 4NS UK; 7 School of Physical Sciences, The Open University, Walton Hall, Milton Keynes, MK7 6AA UK; 8 Institute of Vertebrate Paleontology and Paleoanthropology, 142 Xizhimenwai St., 100044 Beijing, China

## Abstract

Feathers are remarkable evolutionary innovations that are associated with complex adaptations of the skin in modern birds. Fossilised feathers in non-avian dinosaurs and basal birds provide insights into feather evolution, but how associated integumentary adaptations evolved is unclear. Here we report the discovery of fossil skin, preserved with remarkable nanoscale fidelity, in three non-avian maniraptoran dinosaurs and a basal bird from the Cretaceous Jehol biota (China). The skin comprises patches of desquamating epidermal corneocytes that preserve a cytoskeletal array of helically coiled α-keratin tonofibrils. This structure confirms that basal birds and non-avian dinosaurs shed small epidermal flakes as in modern mammals and birds, but structural differences imply that these Cretaceous taxa had lower body heat production than modern birds. Feathered epidermis acquired many, but not all, anatomically modern attributes close to the base of the Maniraptora by the Middle Jurassic.

## Abstract (web summary)

In addition to the evolutionary innovation of feathers, bird skin has complex adaptations. Here, McNamara and colleagues examine exceptionally preserved skin from feathered dinosaurs and ancient birds from the Cretaceous and show the early acquisition of many skin attributes seen in modern species.

## Introduction

The integument of vertebrates is a complex multilayered organ with essential functions in homoeostasis, resisting mechanical stress and preventing pathogenic attack^1^. Its evolution is characterised by recurrent anatomical innovation of novel tissue structures (e.g., scales, feathers and hair) that, in amniotes, are linked to major evolutionary radiations^2^. Feathers are associated with structural, biochemical and functional modifications of the skin^2^, including a lipid-rich corneous layer characterised by continuous shedding^3^. Evo-devo studies^4^ and fossilised feathers^5–7^ have illuminated aspects of early feather evolution, but how the skin of basal birds and feathered non-avian dinosaurs evolved in tandem with feathers has received little attention. Like mammal hair, the skin of birds is thinner than in most reptiles and is shed in millimetre- scale flakes (comprising shed corneocytes, i.e., terminally differentiated keratinocytes), not as large patches or a whole skin moult^2^. Desquamation of small patches of corneocytes, however, also occurs in crocodilians and chelonians and is considered primitive to synchronised cyclical skin shedding in squamates^8^. Crocodilians and birds, the groups that phylogenetically bracket non-avian dinosaurs, both possess the basal condition; parsimony suggests that this skin shedding mechanism was shared with non-avian dinosaurs.

During dinosaur evolution, the increase in metabolic rate towards a true endothermic physiology (as in modern birds) was associated with profound changes in integument structure^9^ relating to a subcutaneous hydraulic skeletal system, an intricate dermo-subcutaneous muscle system, and a lipid-rich corneous layer characterised by continuous shedding^3^. The pattern and timing of acquisition of these ultrastructural skin characters, however, is poorly resolved and there is no a priori reason to assume that the ultrastructure of the skin of feathered non-avian dinosaurs and early birds would have resembled that of their modern counterparts. Dinosaur skin is usually preserved as an external mould^10^ and rarely as organic remains^11,12^ or in authigenic minerals^13–15^. Although mineralised fossil skin can retain (sub-)cellular anatomical features^16,17^, dinosaur skin is rarely investigated at the ultrastructural level^14^. Critically, despite reports of preserved epidermis in a non-feathered dinosaur^10^ there is no known evidence of the epidermis^18^ in basal birds or of preserved skin in feathered non-avian dinosaurs. The coevolutionary history of skin and feathers is therefore largely unknown.

Here we report the discovery of fossilised skin in the feathered non-avian maniraptoran dinosaurs *Beipiaosaurus, Sinornithosaurus* and *Microraptor*, and the bird *Confuciusornis* from the Early Cretaceous Jehol biota (NE China; Supplementary Fig. 1). The ultrastructure of the preserved tissues reveals that feathered skin had evolved many, but not all, modern attributes by the origin of the Maniraptora in the Middle Jurassic.

## Results and discussion

### Fossil soft tissue structure

**Fig. 1:**
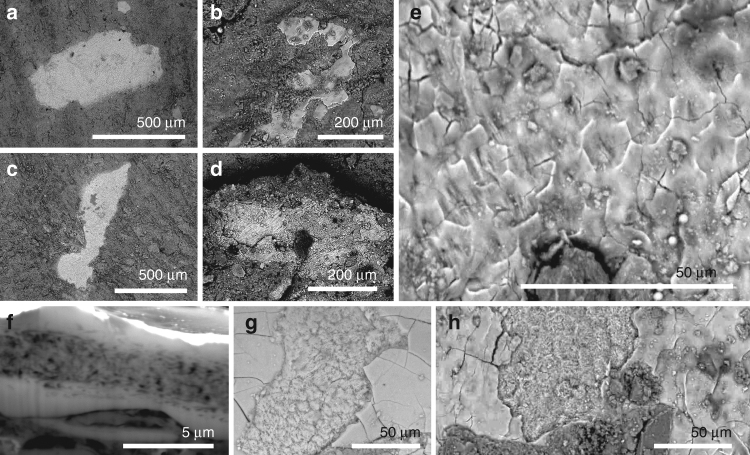
Phosphatised soft tissues in non-avian maniraptoran dinosaurs and a basal bird. **a**–**h** Backscatter electron images of tissue in *Confuciusornis* (IVPP V 13171; **a**, **e**, **f**), *Beipiaosaurus* (IVPP V STM31-1; **b**, **g**), *Sinornithosaurus* (IVPP V 12811; **c**, **h**) and *Microraptor* (IVPP V 17972A; **d**). **a**–**d** Small irregularly shaped patches of tissue. **e** Detail of tissue surface showing polygonal texture. **f** Focused ion beam-milled vertical section through the soft tissue showing internal fibrous layer separating two structureless layers. **g**, **h** Fractured oblique section through the soft tissues, showing the layers visible in **f**

Small patches of tissue (0.01–0.4 mm^2^; Fig. 1a–d and Supplementary Figs. 2–6) are closely associated with fossil feathers (i.e., usually within 500 µm of carbonaceous feather residues, Supplementary Fig. 2e, g, j, k, o, s, t). The patches are definitively of fossil tissue, and do not reflect surface contamination with modern material during sample preparation, as they are preserved in calcium phosphate (see 'Taphonomy', below); further, several samples show margins that are overlapped, in part, by the surrounding matrix. The tissues have not, therefore, simply adhered to the sample surface as a result of contamination from airborne particles in the laboratory.

The tissue patches are typically 3–6 µm thick and planar (Fig. 1a–e). Transverse sections and fractured surfaces show an inner fibrous layer (1.0–1.2 µm thick) between two thinner structureless layers (0.2–0.5 µm thick) (Fig. 1f–h). The external surface of the structureless layer is smooth and can show a subtle polygonal texture defined by polygons 10–15 µm wide (Fig. 1e, h).

**Fig. 2:**
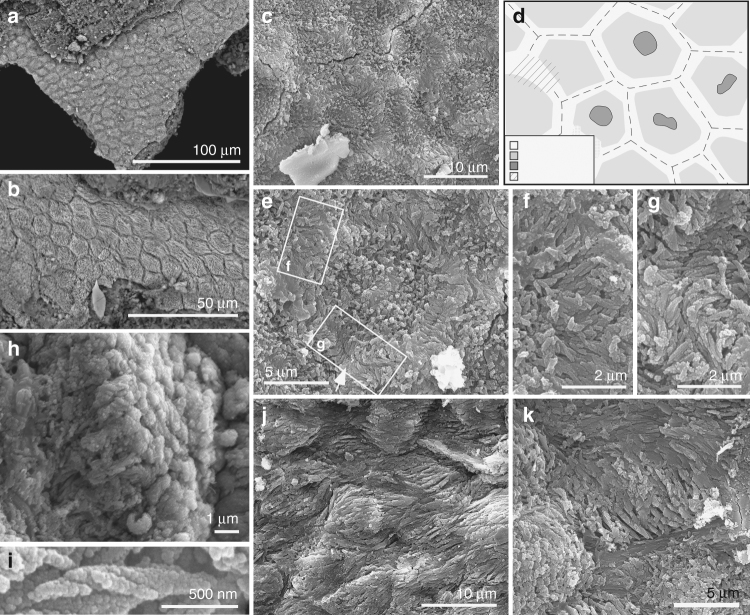
Ultrastructure of the soft tissues in *Confuciusornis* (IVPP V 13171). **a**, **b** Backscatter electron micrographs; all other images are secondary electron micrographs. **a**, **b** Closely packed polygons. **c** Detail of polygons showing fibrous contents, with **d** interpretative drawing. **e**–**g** Polygon (**e**) with detail of regions indicated showing tonofibrils bridging (**f**) and abutting at (**g**) junction between polygons. **h**, **i** Helical coiling in tonofibrils. **h** Oblique view of polygon with central tonofibrils orientated perpendicular to the polygon surface. **j**, **k** Polygons showing stretching-like deformation

The fibrous layer also shows polygons (Figs. 1f, h and 2a–e, and Supplementary Fig. 6) that contain arrays of densely packed fibres 0.1–0.5 µm wide (Fig. 2f–i and Supplementary Fig. 5f). Well-preserved fibres show helicoidal twisting (Fig. 2h, i). Fibres in marginal parts of each polygon are 0.1–0.3 µm wide and oriented parallel to the tissue surface; those in the interior of each polygon are 0.3–0.5 µm wide and are usually perpendicular to the tissue surface (Fig. 2b, h and Supplementary Fig. S6d). In the marginal 1–2 µm of each polygon, the fibres are usually orthogonal to the lateral polygon margin and terminate at, or bridge the junction between, adjacent polygons (Fig. 2f, g and Supplementary Fig. 6e). The polygons are usually equidimensional but are locally elongated and mutually aligned, where the thick fibres in each polygon are sub-parallel to the tissue surface and the thin fibres, parallel to the polygon margin (Fig. 2j, k and Supplementary Fig. 6g–l). Some polygons show a central depression (Fig. 2c–e and Supplementary Fig. 6a–c) in which the thick fibres can envelop a globular structure 1–2 µm wide (Fig. 2e).

### Fossil corneocytes

**Fig. 3:**
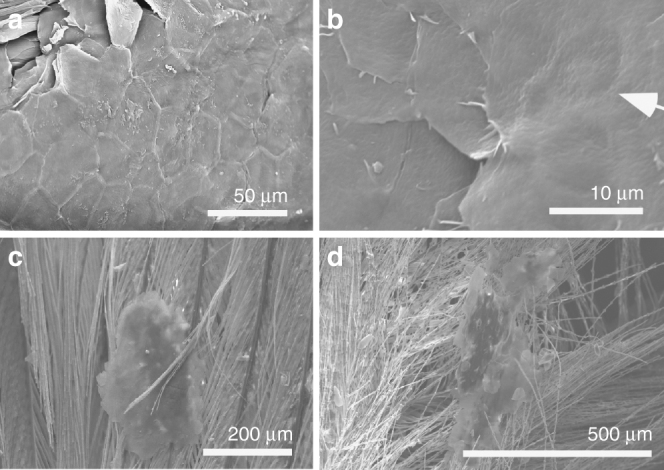
Corneocytes in extant birds. **a**–**d** Scanning electron micrographs of shed skin in extant zebra finch (*Taeniopygia guttata* (*n* = 1); **a**–**d**). **a** Corneocytes defining polygonal texture. **b** Central depression (arrow) marks position of pycnotic nucleus. **c**, **d** Shed skin flakes entrained within feathers

The texture of these fossil tissues differs from that of conchostracan shells and fish scales from the host sediment, the shell of modern *Mytilus*, modern and fossil feather rachis and modern reptile epidermis (Supplementary Fig. 7a–n). The elongate geometry of some polygons (Fig. 2j, k and Supplementary Fig. 6g, l) implies elastic deformation of a non-biomineralized tissue due to mechanical stress. On the basis of their size, geometry and internal structure, the polygonal structures are interpreted as corneocytes (epidermal keratinocytes). In modern amniotes, these are polyhedral-flattened cells (1–3 µm × ca. 15 µm) filled with keratin tonofibrils, lipids and matrix proteins^18–20^ (Fig. 3a, b and Supplementary Figs 2u–x, 8, 9). The outer structureless layer of the fossil material corresponds to the cell margin; it is thicker than the original biological template, i.e., the corneous cell envelope and/or cell membrane, but this is not unexpected, reflecting diagenetic overgrowth by calcium phosphate (see 'Taphonomy'). The fibres in the fossil corneocytes are identified as mineralised tonofibrils: straight, unbranching bundles of supercoiled α-keratin fibrils 0.25–1 µm wide^18,21^ that are the main component of the corneocyte cytoskeleton^22^ and are enveloped by amorphous cytoskeletal proteins^22^. In the fossils, the thin tonofibrils often abut those of the adjacent cell (Fig. 2g and Supplementary Fig. 6e), but locally can bridge the boundary between adjacent cells (Fig. 2f). The latter recalls desmosomes, regions of strong intercellular attachment between modern corneocytes^23^. The central globular structures within the fossil corneocytes resemble dead cell nuclei^24^, as in corneocytes of extant birds (but not extant reptiles and mammals)^24^ (Supplementary Fig. 8). The position of these pycnotic nuclei is often indicated by depressions in the corneocyte surface in extant birds^24^ (Fig. 3b); some fossil cells show similar depressions (Fig. 2c and Supplementary Fig. 6a–c).

### Taphonomy

Keratin is a relatively recalcitrant biomolecule due to its heavily cross-linked paracrystalline structure and hydrophobic nonpolar character^23^. Replication of the fossil corneocytes in calcium phosphate is thus somewhat unexpected as this process usually requires steep geochemical gradients characteristic of early decay^25^ and usually applies to decay-prone tissues, such as muscle^26^ and digestive tissues^27^. Recalcitrant tissues such as dermal collagen can, however, be replicated in calcium phosphate where they contain an inherent source of calcium and, in particular, phosphate ions that are liberated during decay^28^. Corneocytes contain sources of both of these ions. During terminal differentiation, intracellular concentrations of calcium increase^29^ and α-keratin chains are extensively phosphorylated^23^. Further, corneocyte lipid granules^30^ are rich in phosphorus and phosphate^31^. These chemical moieties would be released during degradation of the granules and would precipitate on the remaining organic substrate, i.e., the tonofibrils.

In extant mammals, densely packed arrays of tonofibrils require abundant interkeratin matrix proteins for stability^32^. These proteins, however, are not evident in the fossils. This is not unexpected, as the proteins are rare in extant avian corneocytes^33^ and, critically, occur as dispersed monomers^34^ and would have a lower preservation potential than the highly cross-linked and polymerised keratin bundles of the tonofibrils. The outer structureless layer of the fossil corneocytes is thicker than the likely biological template(s), i.e., the corneous cell envelope (a layer of lipids, keratin and other proteins up to 100 nm thick that replaces the cell membrane during terminal differentiation^34^) and/or cell membrane. This may reflect a local microenvironment conducive to precipitation of calcium phosphate: during terminal differentiation, granules of keratohyalin, an extensively phosphorylated protein^35^ with a high affinity for calcium ions^36^, accumulate at the periphery of the developing corneocytes^37^. The thickness of the outer solid layer of calcium phosphate in the fossils, plus the gradual transition from this to the inner fibrous layer, suggests that precipitation of phosphate proceeded from the margins towards the interior of the corneocytes. In this scenario, phosphate availability in the marginal zones of the cells would have exceeded that required to replicate the tonofibrils. The additional phosphate would have precipitated as calcium phosphate in the interstitial spaces between the tonofibrils, progressing inwards from the inner face of the cell margin.

### Skin shedding in feathered dinosaurs and early birds

In extant amniotes, the epidermal cornified layer is typically 5–20 cells thick (but thickness varies among species and location on the body^38^). The patches of fossil corneocytes, however, are one cell thick (Fig. 1f and Supplementary Figs. 5c, 10). This, plus the consistent small size (<400 μm) of the patches and the remarkably high fidelity of preservation, is inconsistent with selective preservation of a continuous sheet of in situ tissue. In a minority (*n* = 8) of examples, the skin occurs at the edge of the sample of fossil soft tissues and thus could potentially represent a smaller fragment of an originally larger piece of fossil skin (with the remainder of the piece on the fossil slab). In most examples, however, the entire outline of the skin fragment is contained within the margin of a sample. Examination of the margins of various samples at high magnification reveals that the sample and surrounding sediment are often in exactly the same plane (e.g., Supplementary Fig. 10). Even where the margin of the sample of skin is covered by sediment, the sample is unlikely to have been much bigger than the apparent size as the fossil skin, being almost perfectly planar, forms a natural plane of splitting.

There is no evidence that the preserved thickness of the skin is an artefact of preparation or erosion. During splitting of a rock slab, the plane of splitting frequently passes through the soft tissues in an uneven manner, exposing structures at different depths. In the fossils studied here, the plane of splitting usually passes through the corneocytes (exposing their internal structure), and rarely along the outer face of the corneocyte layer. There is no evidence for removal of more than one layer of corneocytes: FIB sections show preservation of only one layer and several SEM images show complete vertical sections through the preserved skin (where the relationship with the over- and underlying sediment is visible), with evidence for only a single layer of corneocytes. The fibrous internal fill of the fossil corneocytes is exposed where the plane of splitting of the fossil slab passes through the patches of tissue. The topography of the fossil corneocytes, however, varies with the position of the plane of splitting, which can vary locally through the soft tissues on a millimetre scale: the corneocytes can present with raised margins and a central depression, or with depressed margins and a central elevated zone (Fig. S9).

The size, irregular geometry and thickness of the patches of corneocytes resemble shed flakes of the cornified layer (dandruff-like particles^39^; Fig. 3). In extant birds, corneocytes are shed individually or in patches up to 0.5 mm^2^ that can be entrained within feathers (Fig. 3c, d and Supplementary Fig. 2u, v). The fossils described herein provide the first evidence for the skin shedding process in basal birds and non-avian maniraptoran dinosaurs and confirm that at least some non-avian dinosaurs shed their skin in small patches^40^. This shedding style is identical to that of modern birds^18^ (Fig. 3c, d) and mammals^20^ and implies continuous somatic growth. This contrasts with many extant reptiles, e.g., lepidosaurs, which shed their skin whole or in large sections^21^, but shedding style can be influenced by factors such as diet and environment^41^.

### Evolutionary implications of fossil corneocyte structure

The fossil corneocytes exhibit key adaptations found in their counterparts in extant birds and mammals, especially their flattened polygonal geometry and fibrous cell contents consistent with α-keratin tonofibrils^16^. Further, the fossil tonofibrils (as in extant examples^22^) show robust intercellular connections and form a continuous scaffold across the corneocyte sheet (Fig. 2b, c, j and Supplementary Fig. 6). In contrast, corneocytes in extant reptiles contain a homogenous mass of β-keratin (with additional proteins present in the cell envelope) and fuse during development, forming mature β-layers without distinct cell boundaries^42^. The retention of pycnotic nuclei in the fossil corneocytes is a distinctly avian feature not seen in modern reptiles (but see ref. ^20^).

**Fig. 4:**
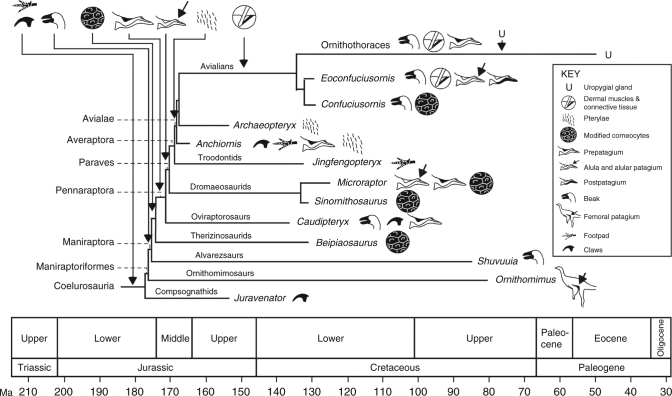
Schematic phylogeny, scaled to geological time, of selected coelurosaurs showing the pattern of acquisition of key modifications of the skin. The phylogeny is the most likely of the maximum likelihood models, based on minimum-branch lengths (mbl) and transitions occurring as all-rates-different (ARD). Claws and footpads are considered primitive in coelurosaurs. Available data indicate that modified keratinocytes, and continuous shedding, originated close to the base of the Maniraptora; this is predicted to shift based on future fossil discoveries towards the base of the Coelurosauria to include other feathered taxa

Epidermal morphogenesis and differentiation are considered to have diverged in therapsids and sauropsids^31^. Our data support other evidence that shared epidermal features in birds and mammals indicate convergent evolution^43^ and suggest that lipid-rich corneocyte contents may be evolutionarily derived characters in birds and feathered non-avian maniraptorans. Evo-devo studies have suggested that the avian epidermis could have arisen from the expansion of hinge regions in ‘protofeather’-bearing scaly skin^20^. While fossil evidence for this transition is lacking, our data show that the epidermis of basal birds and non-avian maniraptoran dinosaurs had already evolved a decidedly modern character, even in taxa not capable of powered flight. This does not exclude the possibility that at least some of the epidermal features described here originated in more basal theropods, especially where preserved skin lacks evidence of scales (as in *Sciurumimus*^44^). Refined genomic mechanisms for modulating the complex expression of keratin in the epidermis^45^, terminal differentiation of keratinocytes and the partitioning of α- and β-keratin synthesis in the skin of feathered animals^32^ were probably modified in tandem with feather evolution close to the base of the Maniraptora by the late Middle Jurassic (Fig. 4). Existing fossil data suggest that this occurred after evolution of the beak in Maniraptoriformes and before evolution of the forelimb patagia and pterylae (Fig. 4); the first fossil occurrences of all of these features span ca. 10–15 Ma, suggesting a burst of innovation in the evolution of feathered integument close to and across the Lower-Middle Jurassic boundary. The earliest evidence for dermal musculature associated with feathers is ca. 30 Ma younger, in a 125 Ma ornithothoracean bird^17^. Given the essential role played by this dermal network in feather support and control of feather orientation^18^, its absence in feathered non-avian maniraptorans may reflect a taphonomic bias.

In certain aspects, the fossil corneocytes are distinctly non-avian and indicate that feathered dinosaurs and early birds had a unique integumentary anatomy and physiology transitional between that of modern birds and non-feathered dinosaurs. In extant birds, corneocyte tonofibrils are dispersed loosely among intracellular lipids^19^; this facilitates evaporative cooling in response to heat production during flight and insulation by plumage^46^. In contrast, the fossil tonofibrils are densely packed and fill the cell interior. There is no evidence for post-mortem shrinkage of the fossil corneocytes: the size range is consistent with those in modern birds, and there is no evidence for diagenetic wrinkling, contortion or separation of individual cells. This strongly suggests that the preserved density of tonofilaments in the fossil corneocytes reflects originally higher densities than in extant birds. This is not a function of body size: extant birds of disparate size (e.g., zebra finch and ostrich) exhibit loosely dispersed tonofibrils^47^. The fossil birds are thus likely to have had a lower physiological requirement for evaporative cooling and, in turn, lower body heat production related to flight activity^46^ than in modern birds. This is consistent with other evidence for low basal metabolic rates in non-avian maniraptoran dinosaurs^47,48^ and basal birds^47^ and with hypotheses that the feathers of *Microraptor*^49^ and, potentially, *Confuciusornis*^48^ (but see ref. ^50^) were not adapted for powered flight, at least for extended periods^50^.

## Methods

### Fossil material

This study used the following specimens in the collections of the Institute for Vertebrate Palaeontology and Paleanthropology, Beijing, China: *Confuciusornis* (IVPP V 13171), *Beipiaosaurus* (IVPP V STM31-1), *Sinornithosaurus* (IVPP V 12811) and *Microraptor* (IVPP V 17972A). Small (2–10 mm^2^) chips of soft tissue were removed from densely feathered regions of the body during initial preparation of the specimens and stored for later analysis. Precise sampling locations are not known.

### Modern bird tissues

Male specimens of the zebra finch *Taeniopygia guttata* (*n* = 1) and the Java sparrow *Lonchura oryzivora* (*n* = 2) were euthanased via cervical dislocation. Individual feathers dissected from *T. guttata* and moulted down feathers from a male specimen of the American Pekin duck (*Anas platyrhynchos domestica*) were not treated further. Small (ca. 10–15 mm^2^) pieces of skin and underlying muscle tissue were dissected from the pterylae of the breast of reproductively active male specimens of *L. oryzivora* raised predominantly on a diet of seeds in October 2016. Tissue samples were fixed for 6 h at 4 °C in 4% paraformaldehyde. After snap freezing in isopentane, tissue was coronal sectioned (10 µm thickness) with a Leica CM1900 cryostat. All sections were allowed to air dry at room temperature for 3 h and stored at −80 °C prior to immunohistology.

### Ethics

The authors have complied with all relevant ethical regulations. Euthanasia of *T. guttata* and *L. oryzivora* was approved by the Health Products Regulatory Authority of Ireland via authorisation AE19130-IO87 (for *T. guttata*) and CRN 7023925 (for *L. oryzivora*).

### Electron microscopy

Samples of soft tissues were removed from fossil specimens with sterile tools, placed on carbon tape on aluminium stubs, sputter coated with C or Au and examined using a Hitachi S3500-N and a FEI Quanta 650 FEG SEM at accelerating voltages of 5–20 kV.

Untreated feathers and fixed and dehydrated samples of skin from modern birds were placed on carbon tape on aluminium stubs, sputter coated with C or Au and examined using a Hitachi S3500-N and a FEI Quanta 650 FEG SEM at accelerating voltages of 5–20 kV. Selected histological sections of *L. oryzivora* were deparaffinized in xylene vapour for 3 × 5 min, sputter coated with Au, and examined using a FEI Quanta 650 FEG SEM at an accelerating voltage of 15 kV. The brightness and contrast of some digital images were adjusted using Deneba Canvas software.

### Focussed ion beam-scanning electron microscopy

Selected samples of fossil tissue were analysed using an FEI Quanta 200 3D FIB-SEM. Regions of interest were coated with Pt using an in situ gas injection system and then milled using Ga ions at an accelerating voltage of 30 kV and a beam current of 20 nA–500 pA.

### Immunohistology

Histological sections were incubated in permeabilization solution (0.2% Triton X-100 in 10 mM phosphate-buffered saline (PBS)) for 30 min at room temperature, washed once in 10 mM PBS and blocked in 5% normal goat serum in 10 mM PBS for 1 h at room temperature. Sections were incubated in primary antibody to cytokeratin (1:300; ThermoFisher) in 2% normal goat serum in 10 mM PBS overnight at 4 °C. Following. 3 × 5 min wash in 10 mM PBS, sections were incubated with a green fluorophore-labelled secondary antibody (1:500; Invitrogen) for 2 h at room temperature. After a 3 × 10 min wash in 10 mM PBS, sections were incubated in BisBenzimide nuclear counterstain (1:3000; Sigma-Aldrich) for 4 min at room temperature. Sections were washed briefly, mounted and coverslipped with PVA-DABCO.

### Confocal microscopy

Digital images were obtained using an Olympus AX70 Provis upright fluorescence microscope and a ×100 objective and stacked using Helicon Focus software (www.heliconfocus.com).

### Data availability

The data that support the findings of this study can be downloaded from the CORA repository (www.cora.ucc) at http://hdl.handle.net/10468/5795.

## Electronic supplementary material

Supplementary Information
