# Supplementary material for: Fossilized skin reveals coevolution with feathers and metabolism in feathered dinosaurs and early birds
Source: Nat Commun. 2018 May 25;9:2072. doi: 10.1038/s41467-018-04443-x (PMC5970262; doi:10.1038/s41467-018-04443-x)
Supplement: Supplementary file 1 — Supplementary Information [file 41467_2018_4443_MOESM1_ESM.pdf]

Supplementary Information for

Fossilized skin reveals coevolution with feathers and metabolism in feathered dinosaurs and early birds

McNamara et al.

Supplementary Figures 1–10

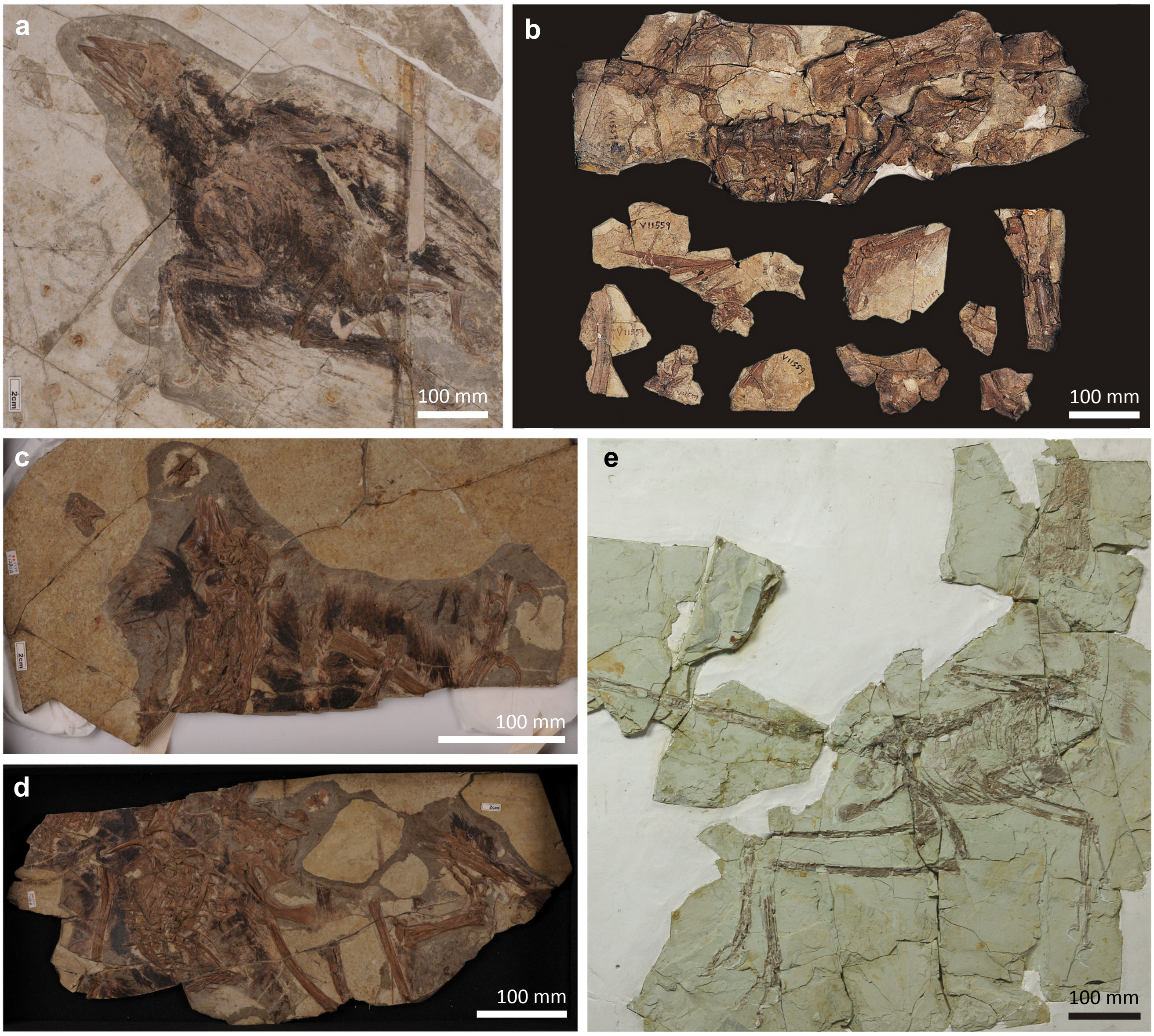

Supplementary Figure 1. Studied specimens. a *Confuciusornis* (IVPP V13171). **b** *Beipiaosaurus* (IVPP V11559). **c, d** *Sinornithosaurus* (IVPP V12811). **e** *Microraptor* (IVPP V17972).

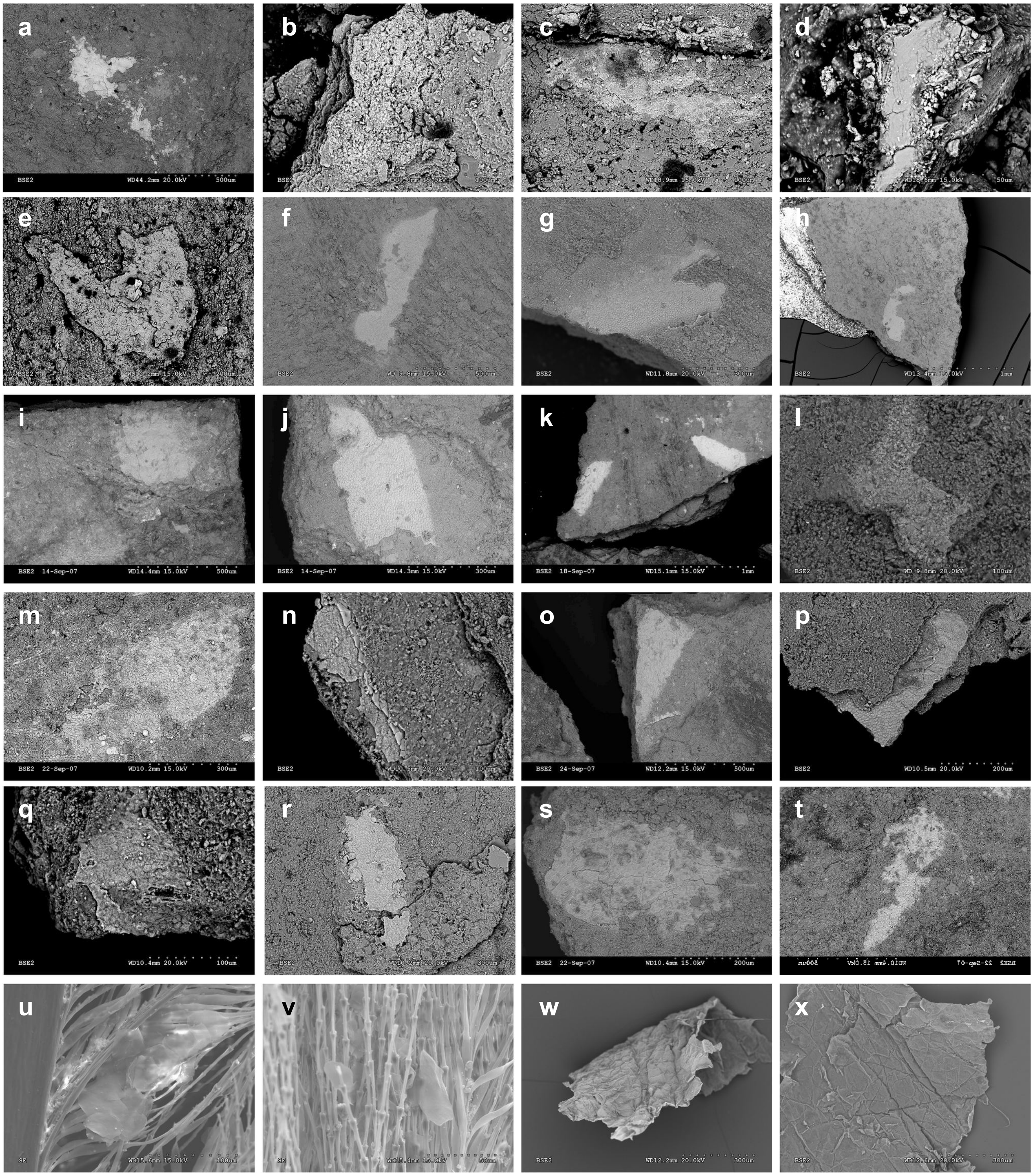

Supplementary Figure 2. Electron micrographs of epidermis in fossils and extant birds. a-t Representative backscatter electron micrographs of patches of epidermis in the feathered dinosaurs *Beipiaosaurus* (**a**), *Microraptor* (**b–d**) and *Sinornithosaurus* (**e–h**) and the fossil bird *Confuciusornis* (**i–t**) confirm consistent preservation of skin in small irregular regions. **u–x** Scanning electron micrographs of dandruff in feathers from the zebra finch (*Taeniopygia guttata*; **u**, **v**) and American Pekin duck (*Anas platyrhynchus domestica*; **w**, **x**).

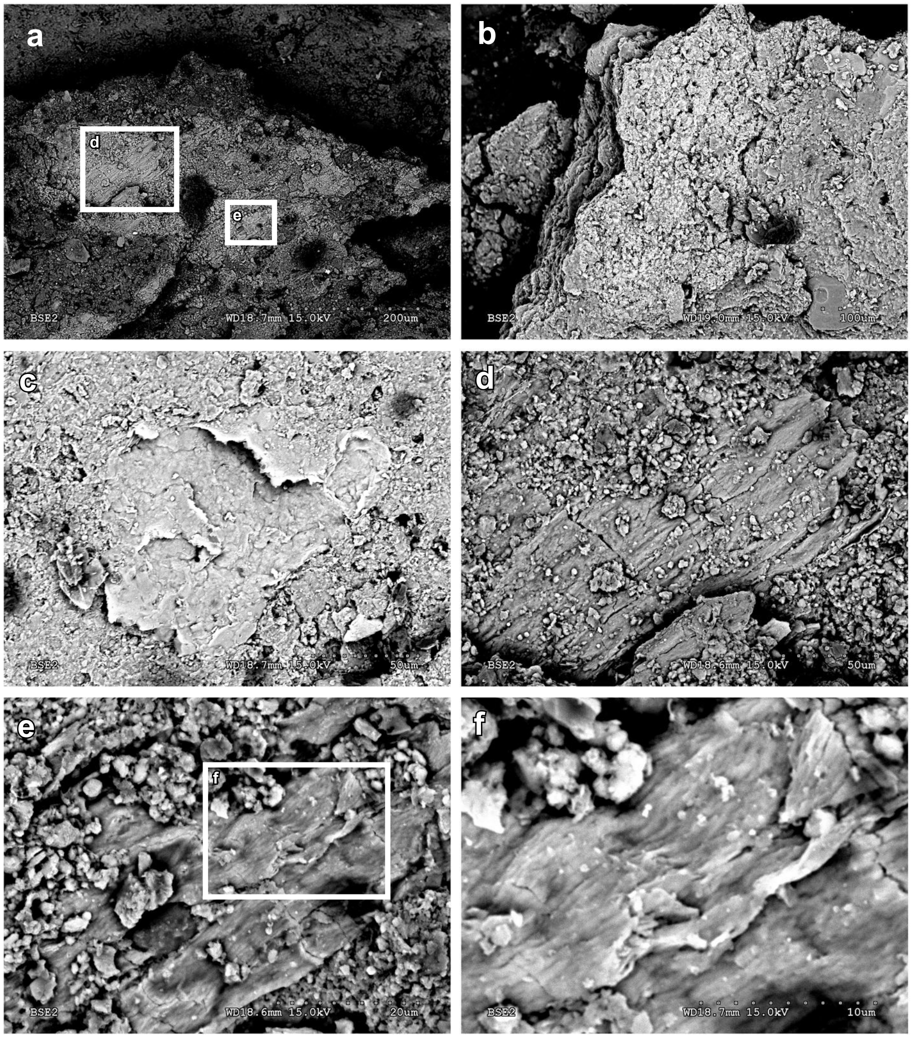

Supplementary Figure 3. Backscatter electron micrographs of epidermis in *Microraptor* (IVPP V 17972A).

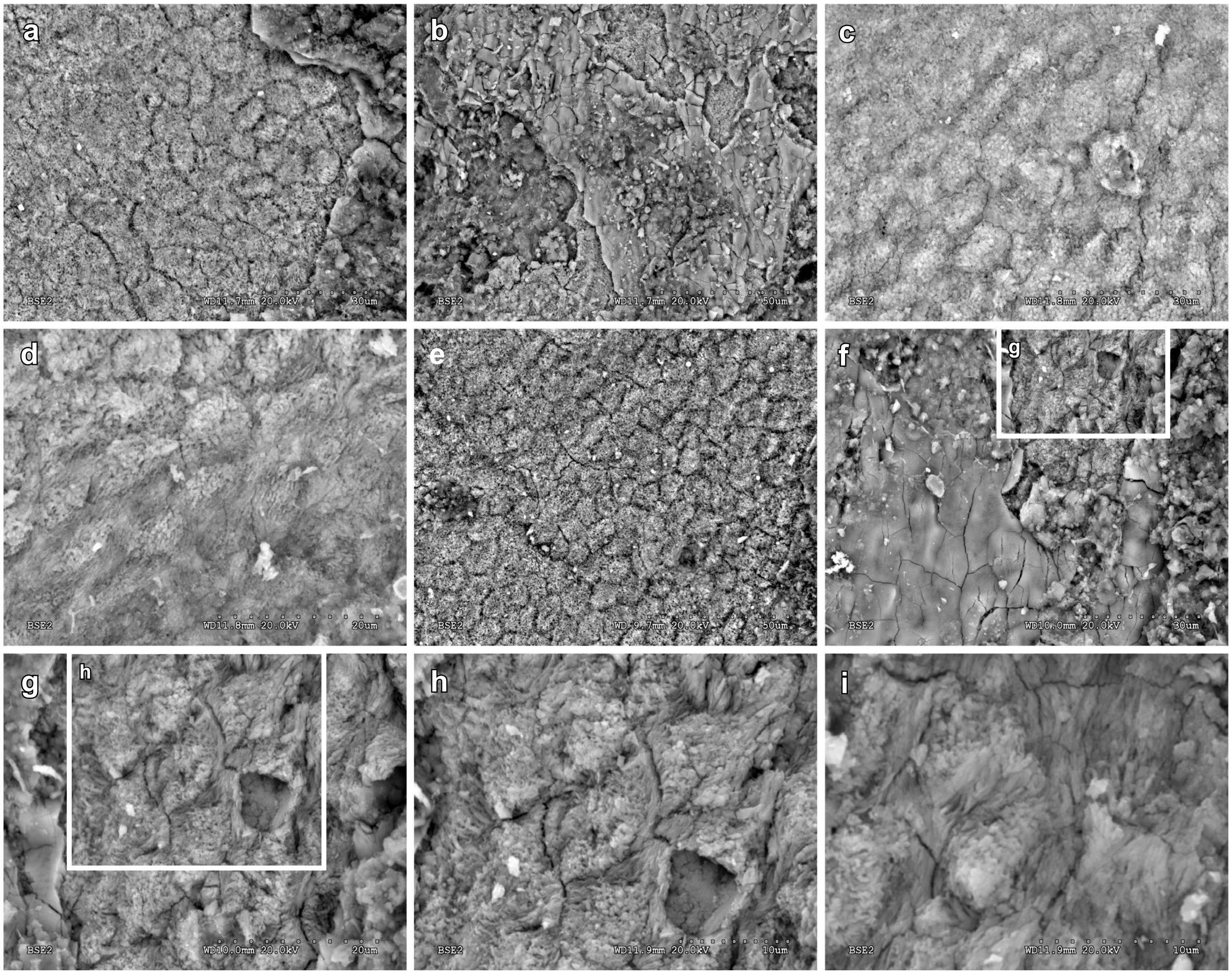

Supplementary Figure 4. Backscatter electron micrographs of phosphatized epidermis in *Sinornithosaurus* (IVPP V 12811). Note polygons in **a**, **c–e**, layered structure in **b**, **f**, and details of fibrous inner layer in **h**, **i**.

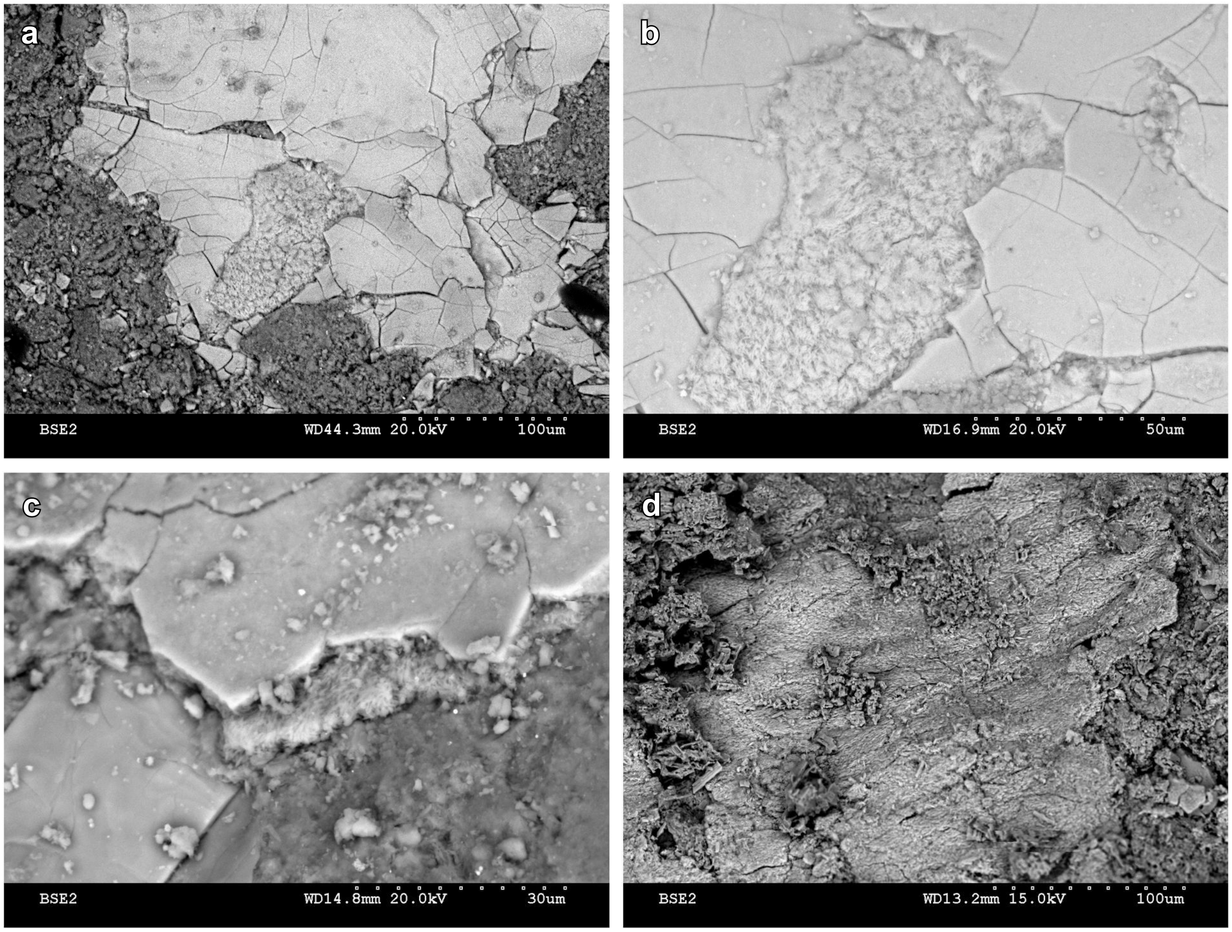

Supplementary Figure 5. Backscatter electron micrographs of phosphatized epidermis in *Beipiaosaurus* (IVPP V STM31-1). Note polygons in **a**, **b**, layered structure in **a**–**c**, and fibrous inner layer in **c**, **d**.

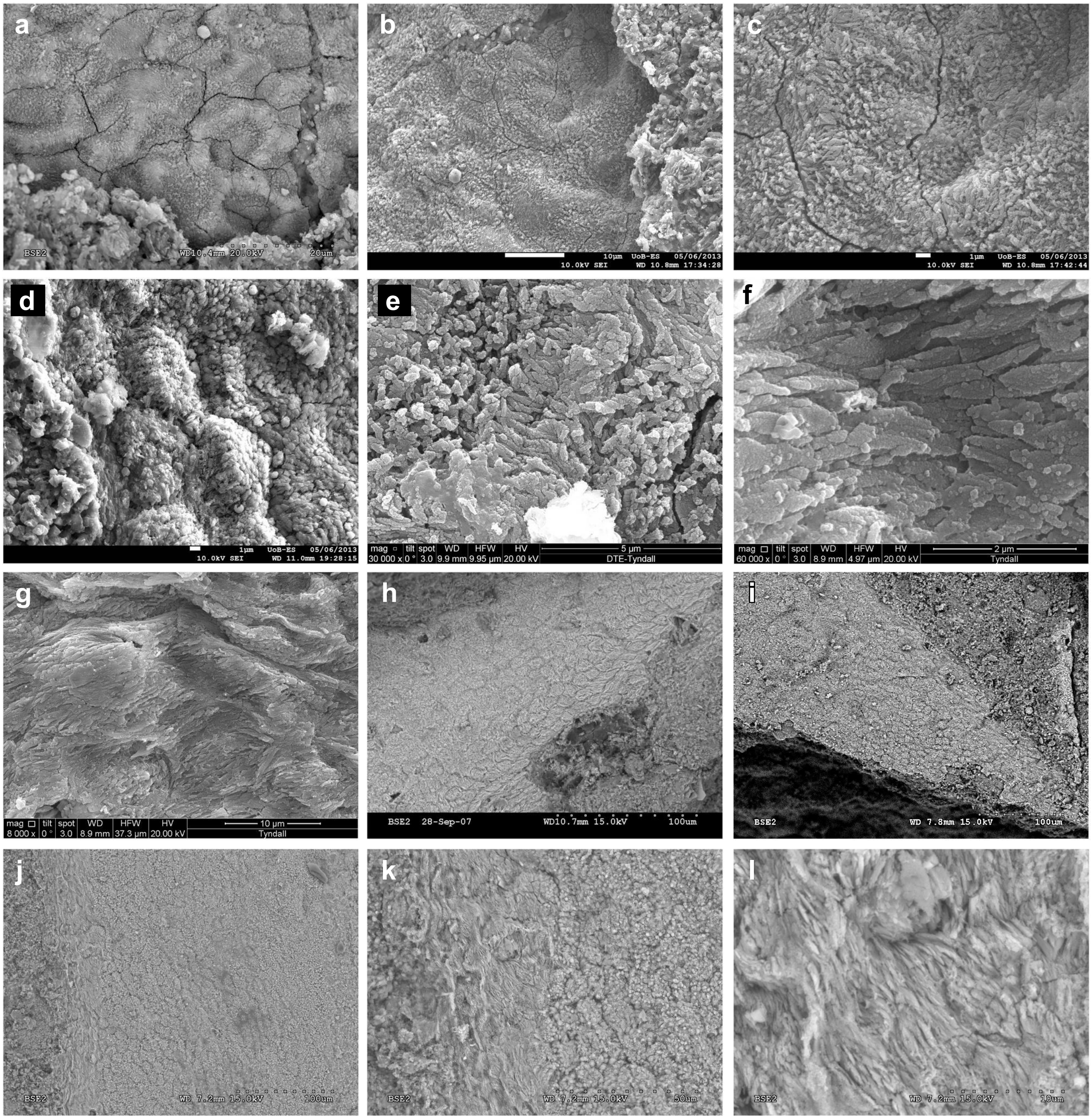

Supplementary Figure 6. Backscatter (a, h–l) and scanning (b-g) electron micrographs of phosphatized epidermal corneocytes in *Confuciusornis.* a–c Polygons showing central depressions. **d** Oblique view of polygons showing thick fibres orientated orthogonally to the surface of the polygon layer. **e** Detail of boundary between two polygons showing abutting fibres from adjacent polygons. **f** Detail of fibres. **g** Polygons showing elongate morphology. **h–j** Polygons from three different samples showing gradual transition from regular hexagonal morphologies to deformed, elongate morphologies. **k**, **l** Progressively more detailed views of **j**.

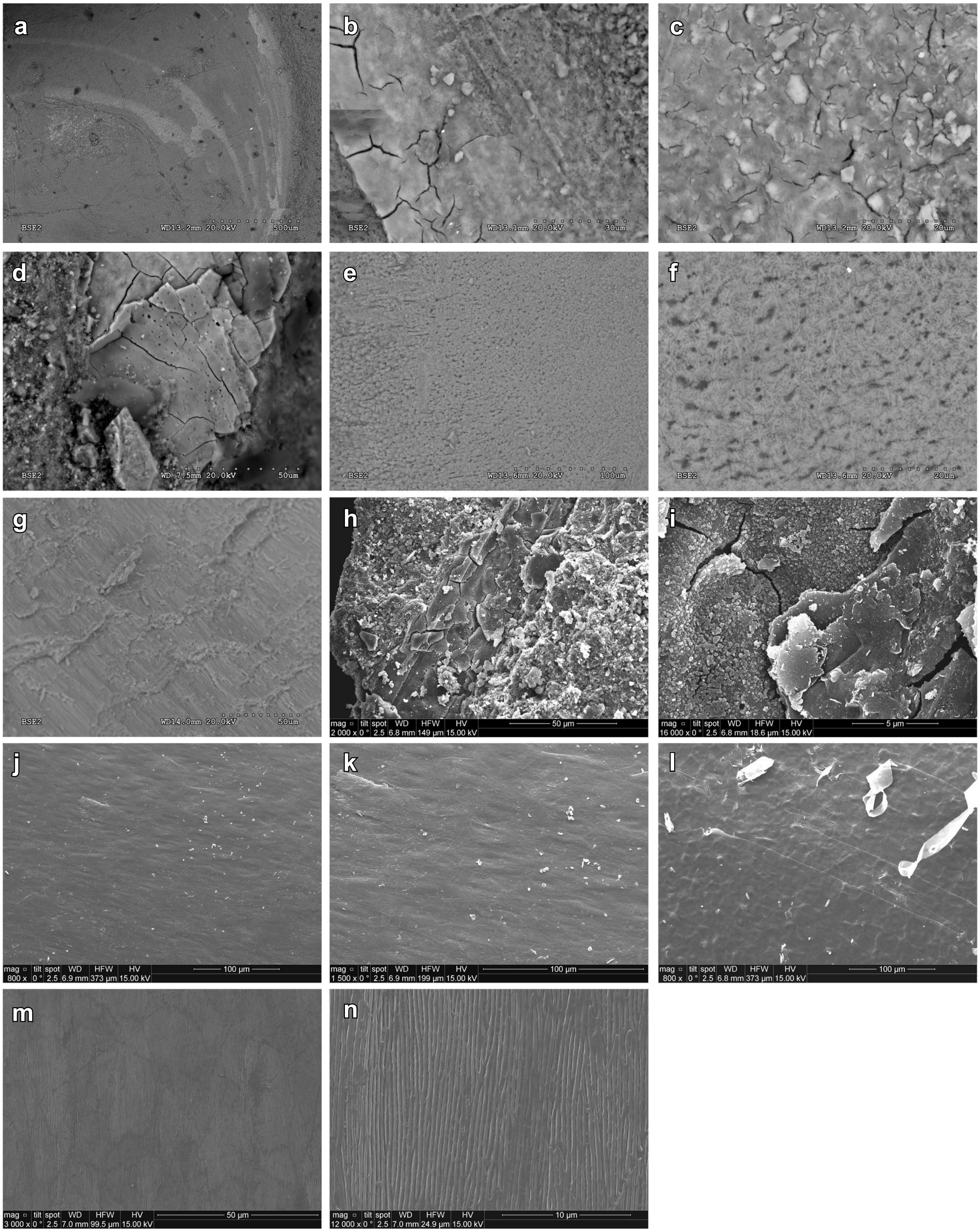

Supplementary Figure 7. Caption overleaf.

Supplementary Figure 7 (previous page). Backscatter electron micrographs of biomineralized tissues in other taxa (a–g) and keratinous tissues in *Confuciusornis* and modern birds (h–n). a–c Fossil conchostrocan shell from Jehol sediments. **d** Unidentified fish scale from Jehol sediments. **e–g** Extant *Mytilus* shell. **h**, **i** Feather rachis preserved in calcium phosphate in *Confuciusornis.* **j–l** Rachis in feather of modern zebra finch *T. guttata*. **m**, **n** Epidermis of the extant snake *Thamnosirtalis sp.*

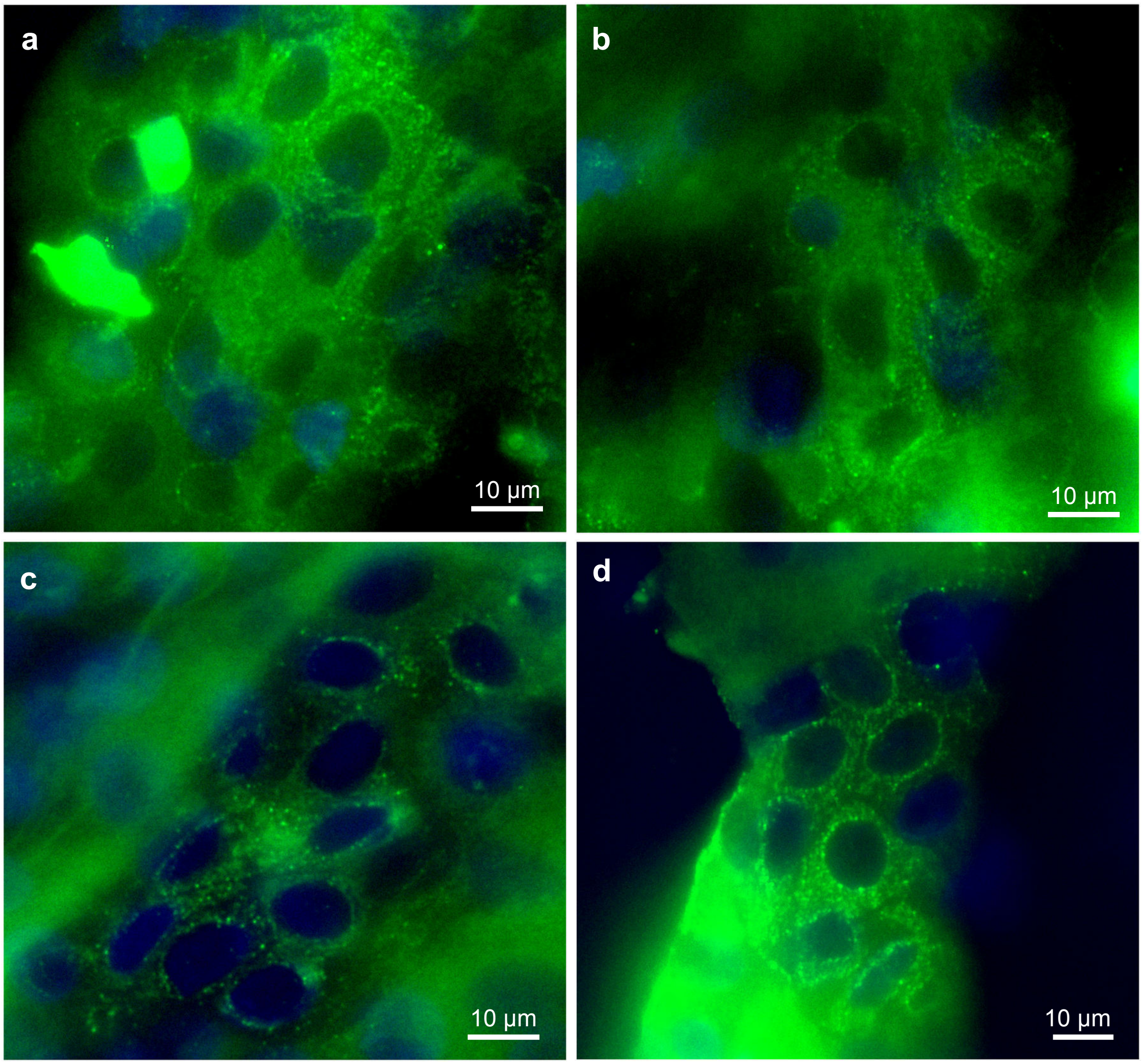

Supplementary Figure 8. Confocal micrographs of cryosections of corneocytes in extant Java sparrow (*Lonchura oryzivora*) stained with a pan-keratin antibody (green) and bisbenzimide (blue). The blue regions correspond to the positions of corneocyte cell nuclei. Although individual keratin tonofibrils cannot be resolved in the cell bodies at this magnification, the latter are clearly rich in keratin.

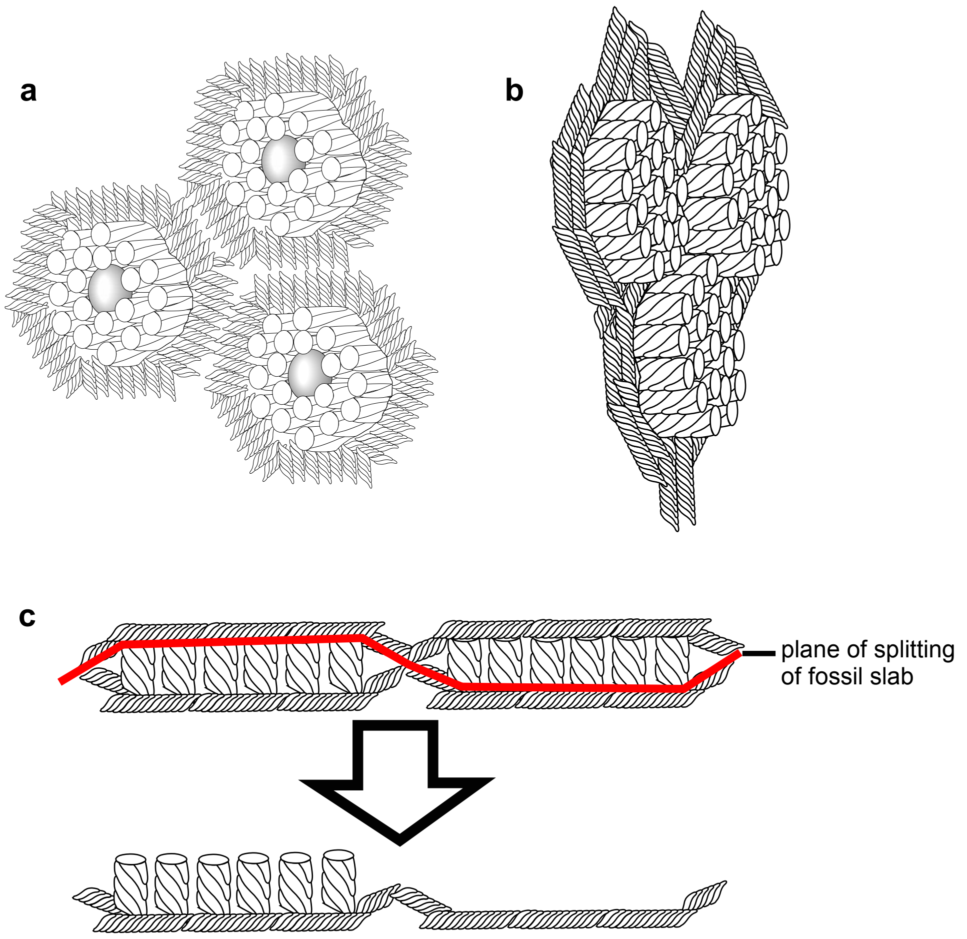

Supplementary Figure 9. Schematic interpretative drawings of the internal structure of the fossil corneocytes showing thick central fibres and thin marginal fibres in each polygon. a Polygons in plan view with thin fibres perpendicular to the polygon margins. Central round feature is pycnotic nucleus. **b** Elongate, deformed polygons in plan view with thin fibres parallel to the polygon margins. **c** Polygons in transverse sectional view showing the impact of the plane of splitting upon the appearance, especially topography, of the polygons. On the left, the plane of splitting produces polygons that present with central regions as topographic highs and margins as topographic lows, e.g. **Fig. 2a**, **b**, **g**, **i**; **Fig. S5d**. On the right, the plane of splitting produces polygons that present with central regions as topographic lows and margins as topographic highs, e.g. **Fig. 2c**; **Fig. S5a–c**.

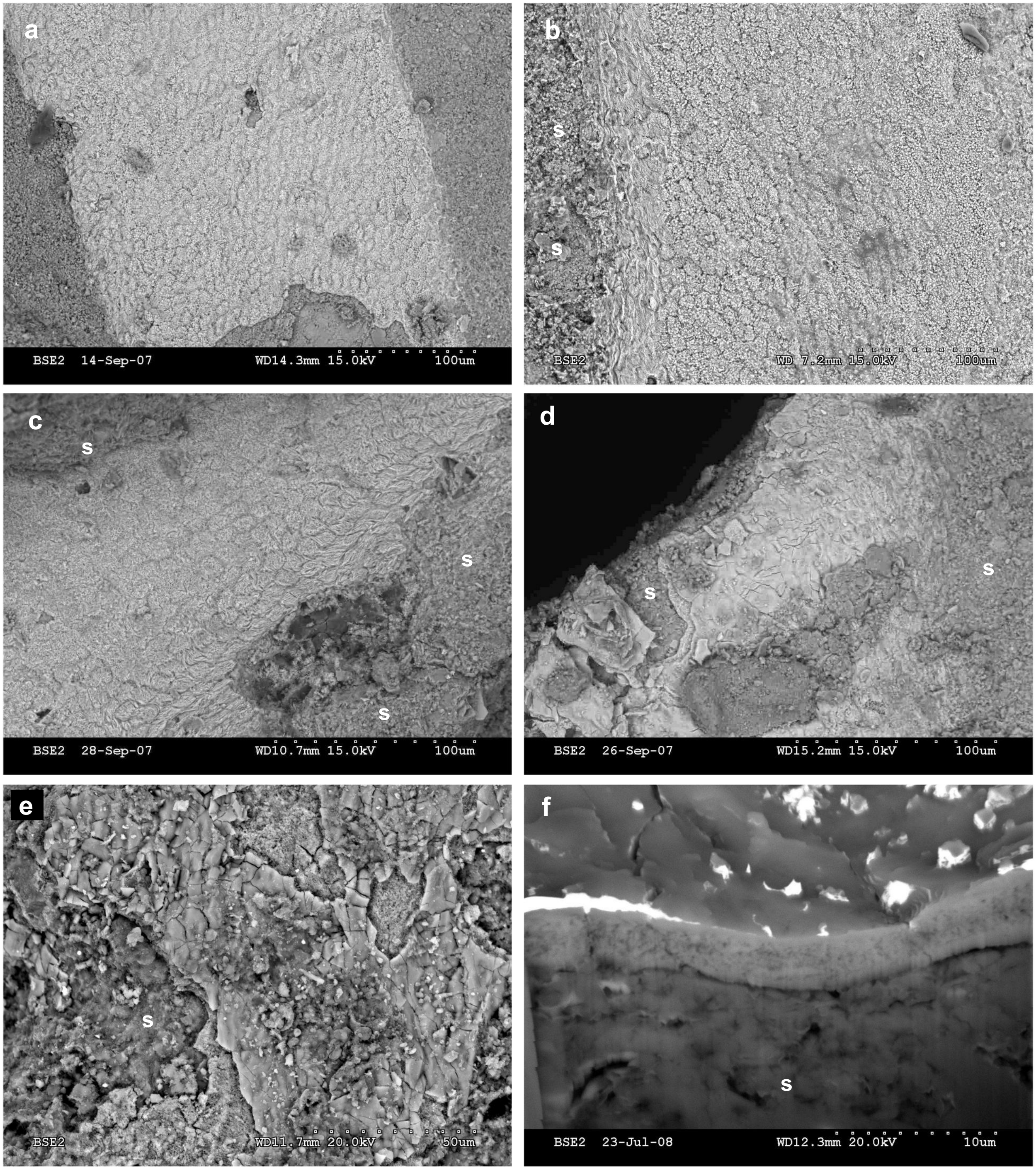

Supplementary Figure 10. Backscatter electron micrographs of phosphatized epidermal corneocytes in *Confuciusornis* (a–d, f) and *Sinornithosaurus* (e). a–d Corneocyte layer is in the same plane as the sediment (to both sides of the sample in **a**, to the right-hand side of the sample in **b–d**). **d–f** Exposure of the entire thickness of the fossil skin, showing both upper and lower surfaces; external surfaces are smooth and internal surfaces are fibrous, preserving tonofibrils. The preserved skin represents one cell layer; there is no evidence for additional layers. **f** is a focussed ion beam-milled surface.
